# Effect of Metal Additives on the Structure, Morphology, and Adsorption Characteristics of the Composites: Silicon Monoxide/Phenol–Formaldehyde-Derived Carbon

**DOI:** 10.3390/ijms26104770

**Published:** 2025-05-16

**Authors:** Mariia Galaburda, Agnieszka Chrzanowska, Dariusz Sternik, Malgorzata Zienkiewicz-Strzalka, Anna Derylo-Marczewska

**Affiliations:** 1 Institute of Chemical Sciences, Faculty of Chemistry, Maria Curie-Skłodowska University, Maria Curie-Sklodowska Sq.3, 20-031 Lublin, Poland; agnieszka.chrzanowska@mail.umcs.pl (A.C.); dariusz.sternik@mail.umcs.pl (D.S.); malgorzata.zienkiewicz-strzalka@mail.umcs.pl (M.Z.-S.); 2 Chuiko Institute of Surface Chemistry, 17 General Naumov Str., 03164 Kyiv, Ukraine

**Keywords:** carbon nanocomposites, phenol–formaldehyde-derived carbon, adsorption isotherms, organics adsorption

## Abstract

The role of metal additives in the synthesis of composite materials based on the silicon and carbon-containing materials to create the desired structural and adsorption properties is analyzed. A two-step procedure was applied to obtain a series of nanocomposites doped with metal oxides. Various techniques were used to characterize the phase composition and the textural, structural, morphological, and thermal properties of the synthesized materials: X-ray diffraction, scanning electron microscopy, Raman spectroscopy, nitrogen adsorption–desorption, and thermal analysis. The adsorption processes on the obtained nanocomposites were studied for aqueous solutions of aniline, benzoic acid, and phenol. The influence of the metal additives on the formation of carbonaceous structures, the adsorption efficiency, and the adsorption mechanism was determined. The synthesized composites show mesoporous and microporous structures, with varied proportions of both pore types. They are differentiated, taking into account the quality of the carbon material (defect density and degree of graphitization), which decreases in the Co > Ni > Cu > Zn > SiO line. The complex effect of the factors determining the adsorption mechanism and efficiency was investigated: textural, structural, and morphological characteristics and the role of the active metal centers. Generally, the results provide valuable insights into the adaptation of hybrid materials for various industrial applications and underline their versatility.

## 1. Introduction

Environmental degradation caused by industrial and domestic waste production, leading to water pollution, poses a major threat to ecological integrity, as the accumulation of persistent toxic compounds disrupts food webs, degrades habitat quality, and limits biodiversity [1,2]. Among these pollutants, organic compounds are of particular concern due to their widespread distribution and high levels of pollution. Due to their different physiochemical properties, solubility, chemical stability, and toxicity, they represent different classes of compounds, which adds to the problems of their removal from the environment [3]. They mainly enter the aquatic environment via industrial effluents, agricultural runoff, and municipal wastewater, where they can bioaccumulate and cause adverse effects ranging from endocrine disruption and carcinogenicity to acute toxicity in aquatic organisms [4,5]. To mitigate these risks, adsorption has proven to be a versatile, cost-effective treatment strategy that offers high removal efficiency under mild operating conditions [6,7].

New methods and materials are being developed to remove toxic substances, separate mixtures, and recover some of the components. Porous carbon materials (active carbon, graphene, CNTs, etc.) are often used as adsorbents for organic pollutants due to their large surface area, hierarchical pore structure, and surface functional group [8]. Various synthesis methods to produce carbon, including pyrolysis, chemical vapor deposition (CVD), and hydrothermal carbonization, have been used to optimize the structure and functionality of carbon materials for environmental and energy applications [9,10,11]. A wide range of polymeric feedstocks can be pyrolyzed to form carbon adsorbents, from biomass-derived feedstocks (yielding biochar) to synthetic polymers (yielding engineered charcoal). Biochars, produced by the thermal decomposition of biomass (e.g., wood, agricultural waste) at 300–800 °C, have attracted attention as low-cost, sustainable sorbents for organic compounds and often show comparable performance to commercial activated carbons [12,13,14]. On the synthetic side, phenolic resins (phenol–formaldehyde, PF) are a common carbon precursor due to their high carbon yield and aromatic network structure. During pyrolysis (typically 600–1000 °C), the cured PF resin undergoes dehydration and graphitization reactions that transform it into an amorphous carbon matrix [15,16,17]. The resulting carbon often has a glassy structure, rich in microporosity, which can be further activated or graphitized as required. By adjusting the polymer formulation and pyrolysis conditions, a variety of carbon nanoarchitectures (from nanoporous powders to monolithic carbons) can be obtained [16,18].

The addition of additives or fillers during carbonization is an effective strategy to control the structure, porosity, and morphology of the resulting carbon. Transition metal catalysts such as Fe, Co, or Ni are known to catalyze graphitization and lower the temperature required for the formation of graphitic carbon domains and even grow carbon nanotubes or nanofibers in situ [16,19,20,21]. For example, direct pyrolysis of organometallic feedstocks (such as ferrocene or metal-doped polymers) can lead to CNT-rich carbons as the metal nanoparticles drive the formation of tubular graphitic shells [19,22,23]. Such metal-catalyzed carbons usually have higher crystallinity (graphitic or turbostratic structures) and improved electrical conductivity. In contrast, inorganic fillers such as silicon oxides (SiO or SiO_2_) and other removable shells (e.g., salts or metal oxides such as ZnO, MgO) are often added to develop hierarchical porosity. These additives remain inert during pyrolysis but can be etched out afterwards, leaving customized pore networks [24]. For example, nano-silica dispersed in a phenolic resin can form uniform mesopores in the charcoal, while chemical activation with ZnCl_2_ or KOH etches the carbon matrix to create large-surface-area micropores [15,25,26]. By selecting suitable additives, the surface area, pore size distribution, and morphology of the carbon (e.g., particle or monolith) can be adjusted, which in turn influences the adsorption properties.

Metal salts can also create porosity when they decompose or evaporate. For example, ZnCl_2_ serves as an effective activating agent [27]. During pyrolysis, ZnCl_2_ decomposes and volatilizes, promoting the development of micropores and mesopores in the carbon structure. This process not only improves porosity, but also increases the specific surface area of the resulting activated carbon. Surface modifications—both chemical (e.g., oxidation with oxygen or ammonia) and physical—can significantly alter the porosity and specific surface area of activated carbon, thereby increasing the adsorption capacity for hazardous substances by optimizing the surface chemistry and structural properties [28,29]. Overall, metal additives tend to increase micropore volume and surface area. In addition, the metal–carbon interactions (and any resulting oxide/hydroxide phases) create defect sites—including vacancies and oxygen functional groups—which serve as new adsorption sites on the carbon surface. For instance, metal-doped carbons show enhanced defect scattering in Raman spectra compared to untreated carbon, indicating the formation of structural defects [8].

The adsorption of organic molecules from dilute aqueous solutions onto carbonaceous materials occurs through non-electrostatic and electrostatic interactions, which depend on the properties of the adsorbent, the adsorbate, and the solution. In the adsorption of aromatic organic compounds, the hydrophobic and dispersive interactions between the π-electrons of the graphene layers of the adsorbent and those of the benzene ring play an important role. These interactions can be influenced by the functionalization of the adsorbent surface, leading to different adsorption mechanisms: electron donor–acceptor, charge transfer, electrostatic or hydrogen bonding. Numerous studies have investigated porous carbon materials for the adsorptive removal of model organic pollutants such as phenol, aniline, and benzoic acid—compounds that have different functional groups and polarities. Phenol (a weak acid, pKa ≈ 9.9) is often used as a reference pollutant for carbon adsorbents. Activated carbons usually achieve high phenol uptake (in the order of 100–300 mg/g) via π–π dispersion interactions and hydrogen bonding. Mojoudi, N. et al. reported a maximum equilibrium capacity of ~434 mg/g for phenol on a KOH-activated char from oily sludge [30]. They found that phenol removal was strongly correlated with the micropore volume and surface area of the charcoal. Aniline (an aromatic amine, basic with pKa of conjugate acid ≈ 4.6) also adsorbs effectively in the carbon pores. Pardo et al. showed that a range of microporous activated carbons exhibited aniline adsorption capacities ranging from ~139 up to 258 mg/g, which were largely proportional to their BET surface areas and micropore contents [31]. Interestingly, this study found that oxygen-containing surface functional groups (e.g., carboxyl and phenolic groups) can hinder the uptake of aniline at low concentrations, presumably by making the carbon surface more polar and less inclined to interact with neutral aniline molecules. Benzoic acid (a carboxylic acid, pKa ≈ 4.2) presents a different challenge, as it tends to deprotonate near neutral pH. The adsorption of benzoic acid on carbon is strongest in its molecular (undissociated) form and is therefore enhanced at a lower solution pH. Nevertheless, porous carbons can achieve a considerable capacity for benzoic acid. For example, N-doped activated carbons have shown efficient uptake in acidic solutions [32]. It is noteworthy that, as mentioned above, single-walled CNT bundles functionalized with Fe adsorb benzoic acid with a capacity of up to 375 mg/g [33]. Molecular simulations indicate that the bundled graphitic structure provides high-energy binding sites that strongly immobilize benzoic acid and outperform other adsorbents, such as activated carbon cloth or even oxidized multi-walled CNTs. These results show how the physical structure and surface chemistry of carbon adsorbents determine their affinity for different organic molecules.

In summary, the rich diversity of porous carbon materials—ranging from traditional activated carbons to engineered carbon nanostructures—provides a toolbox for removing organic pollutants from water. By selecting appropriate starting materials, additives, and processing methods, carbon adsorbents with tailored porosity and surface characteristics can be developed to remove specific contaminants. The literature shows that the adsorption performance for compounds such as phenol, aniline, and benzoic acid is strongly influenced by the pore architecture and surface functionality of the carbon. The ongoing development of biochar and synthetic carbon frameworks and the strategic use of catalysts and templates further improve adsorption capacity and selectivity. This introduction provides the scientific context and rationale for the present study, which investigates the synthesis of porous carbon materials by polymer pyrolysis with additive modulation and evaluates their effectiveness in adsorbing selected organic pollutants. Each aspect, from material fabrication to the mechanism of the pollutant uptake, builds on the state of the art described above and aims to improve our understanding of carbon-based adsorbents for environmental remediation.

Composites expand the range of porous materials used in numerous applications: carriers, catalysts, adsorbents, and biomedical materials. Depending on the application, porous materials should be characterized by the desired textural, structural, surface, and adsorption properties. The demand for novel single or multi-component materials is the driving force behind research in this field. In particular, materials that comply with the principles of green chemistry are very popular. The main objective of this study was therefore to develop and synthesize novel hybrid carbon composites by combining phenol–formaldehyde polymers with silicon monoxide and incorporating various metals (Co, Cu, Ni, Zn). This was achieved through a two-step process involving mechanochemical mixing followed by pyrolysis at 950 °C in a nitrogen atmosphere. The textural, structural, morphological, thermal, and adsorption properties were determined. The dual role of the metal additive was analyzed: promotion of the structural and textural changes in the materials and influence on the adsorption mechanism and efficiency.

## 2. Results and Discussion

### 2.1. Thermal Decomposition of Polymeric Composites in N_2_ Atmosphere

**Figure 1:**
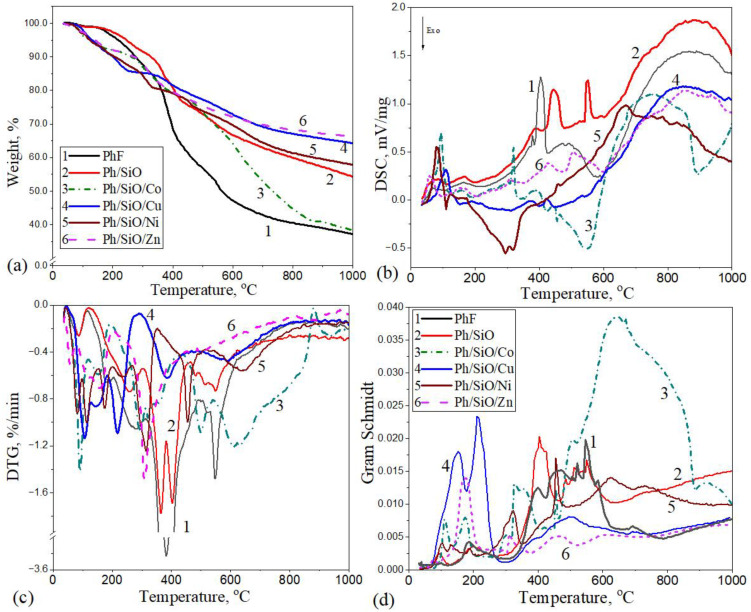
(**a**) TG, (**b**) DSC, (**c**) DTG, and (**d**) Gram–Schmidt curves for PFR (1), PFR/SiO (2), PFR/SiO/Co (3), PFR/SiO/Cu (4), PFR/SiO/Ni (5), and PFR/SiO/Zn (6), measured in N_2_ atmosphere.

Figure 1 shows the TG/DTG/DSC profiles and Gram–Schmidt curves for PF resin and the composites obtained in the first synthesis step before calcination: PFR/SiO, PFR/SiO/Co, PFR/SiO/Cu, PFR/SiO/Ni, and PFR/SiO/Zn. The investigation of the thermal degradation of metal-doped polymer composites in a nitrogen atmosphere (N_2_) is crucial for understanding their thermal stability, their decomposition mechanisms, and their behavior during carbonization. The detailed data for each degradation phase are summarized in Figure 1 and Table 1.

As can be seen from the data obtained, the pyrolysis process is a complex, multistage phenomenon, as shown by the multiple peaks in the DTG and DSC curves in Figure 1. In the first stage (25–150 °C), both the moisture adsorbed on the surface and the intrinsic moisture are released (dehydration). This is followed (partly overlapping with the first stage) by decomposition between 150 and 350 °C, which is mainly due to the degradation of the acetate component in the modified composites [34]. The next stage extends up to 600 °C and corresponds to the thermal degradation of the phenol–formaldehyde resin (PF). In this phase, the polymer matrix begins to decompose as the elevated temperatures break the molecular bonds within the phenol–formaldehyde structure, resulting in the release of volatile compounds. As the temperature increases further, thermal cracking processes dominate, where the decomposition fragments undergo secondary reactions such as condensation and aromatization, eventually leading to the formation of a carbonaceous residue (char).

Thermogravimetric (TG) analysis shows that the PFR alone (Figure 1, curve 1) has the lowest char yield, retaining only ≈37 wt% at 1000 °C. In contrast, the addition of inert SiO filler (PFR/SiO, curve 2) increases the final residue to ≈54 wt%. Among the metal-doped composites, the Zn-containing sample (PFR/SiO/Zn, curve 6) has the highest residual mass (≈67 wt%), closely followed by the Cu sample (PFR/SiO/Cu, curve 4) with ~64 wt%. The Ni sample (PFR/SiO/Ni, curve 5) is intermediate (~58 wt% residual), while the Co sample (PFR/SiO/Co, curve 3) ends up with only ≈39 wt%—essentially the same as pure PFR. The Zn and Cu fillers thus maximize the residue, while Co (despite the added filler) has the lowest char gain. Based on the mass loss in the sample series (Figure 1, TG curves) during pyrolysis at 950 °C, the materials can be ranked in the following order: PFR (63 wt%) > Co (60.4 wt%) > Si (44.2 wt%) > Ni (41.3 wt%) > Cu (34 wt%) > Zn (33.1 wt%). In the case of pure PFR, the yield of pyrolytic oil is higher, and the yield of carbon is lower.

A higher mass loss during pyrolysis usually indicates a stronger decomposition of the polymer precursor. This in turn can lead to a lower yield of carbon material as more of the original material is lost as gasses, meaning less solid carbon remains. On the other hand, it can lead to the formation of denser carbon with high purity. Higher residual masses with metal additions indicate improved char formation, which is useful for applications that require carbon materials.

Differential scanning calorimetry (DSC) and derivative thermogravimetry (DTG) curves (Figure 1b,d) show a multistage decomposition process and provide valuable insights into the thermal behavior of polymers. The temperature profile of the degradation of pure PFR (curve 1) can be seen in a broad temperature range of 300–600 °C, with a sharp endothermic peak at 440 °C. At this temperature, a significant degradation of the polymer chain takes place with the evolution of a series of volatile compounds [35]. This confirms the high thermal stability of the resin. As it is a thermosetting polymer, it does not have a specific melting point. At higher temperatures, the last part of the DSC curves shows additional endothermic behavior, which is due to the formation of carbon-rich residues (cracking process). The SiO-filled resin (curve 2) shows similar peaks at a slightly higher temperature but lower intensity. Silicon monoxide (SiO) is not subject to the typical thermal degradation of organic materials. It is thermally stable and has no decomposition temperature. Therefore, the DSC curve shows no significant exothermic or endothermic peaks related to decomposition.

Metal additions have a significant influence on the DSC and DTG peak intensities and their temperature maxima. The composites with Co and Ni (curves 3 and 5, Table 1) exhibit strong exothermic peaks around 300–600 °C, indicating catalytic effects on carbonization/residue formation and oxidation of methylene in the presence of carbon oxides [36]. Cu as a composite additive (curve 4) delays and broadens the endothermic events, indicating a different interaction mechanism. Zn-containing material (curve 6) shows milder peaks, indicating a lower catalytic activity compared to Co or Ni.

The Gram–Schmidt analysis curve (Figure 1d) captures the cumulative gas evolution during thermal decomposition, which indicates the release of volatile products. As can be seen in Figure 1d, a peak at 350–600 °C of the PFR (curve 1) indicates a larger release of volatile products during the decomposition of the resin. The gas evolution in the PFR/SiO (curve 2) is less intense compared to the pure PFR, reflecting the stabilizing effect of SiO on the decomposition processes and consistent with reports that silica in phenolic compounds increases the formation of fixed carbon [37]. Co- and Ni-containing composites (curves 3 and 5) show multiple intense peaks, indicating catalytic effects that facilitate the degradation of intermediates and the release of volatile gasses. The Cu-doped material (curve 4) promotes the early evolution of volatile organic compounds, potentially shifting the reaction pathways and thus leading to different product distributions. The composite with added Zn (curve 6) is similar to the Cu-containing sample at low temperatures but produces less pronounced peaks at higher temperatures, indicating a lower production of volatile gasses. In general, the metal salts broaden and shift the decomposition stages: all samples still follow the three-stage PFR decomposition (condensation ≲ 450 °C, rapid collapse of the network at 450–700 °C, final dehydrogenation > 700 °C) [38], but the heat flow profiles depend on the catalyst. In summary, Ni and Zn formulations exhibit more gradual gas evolution (consistent with the higher char), while Co and Cu trigger rapid gas emission (consistent with their lower residual mass).

Although the PFR/SiO/Co composite contains both SiO and cobalt, its carbonization yield is not much higher than that of pure PFR. This can be explained by the catalytic activity of cobalt in the polymer matrix. Cobalt species can catalytically oxidize or cleave the –CH_2_– bridges and hydroxymethyl groups of the resin, releasing small molecules (H_2_, CO, CO_2_, H_2_O, etc.; see data below) and thus preventing the formation of solid carbon. However, a complementary SEM analysis (results described below) confirms the formation of carbon nanotubes (CNTs) in this sample, suggesting that cobalt not only promotes the release of volatiles but also catalyzes the restructuring of decomposed carbon species into ordered nanostructures. This dual role—gasification and nanocarbon formation—helps to explain the low residual mass: although some of the solid carbon forms as CNTs, much of the polymer matrix is converted to gaseous products during pyrolysis. The co-additive thus shifts the carbon yield from amorphous charcoal to structurally defined CNTs without significantly increasing the total residue.

**Figure 2:**
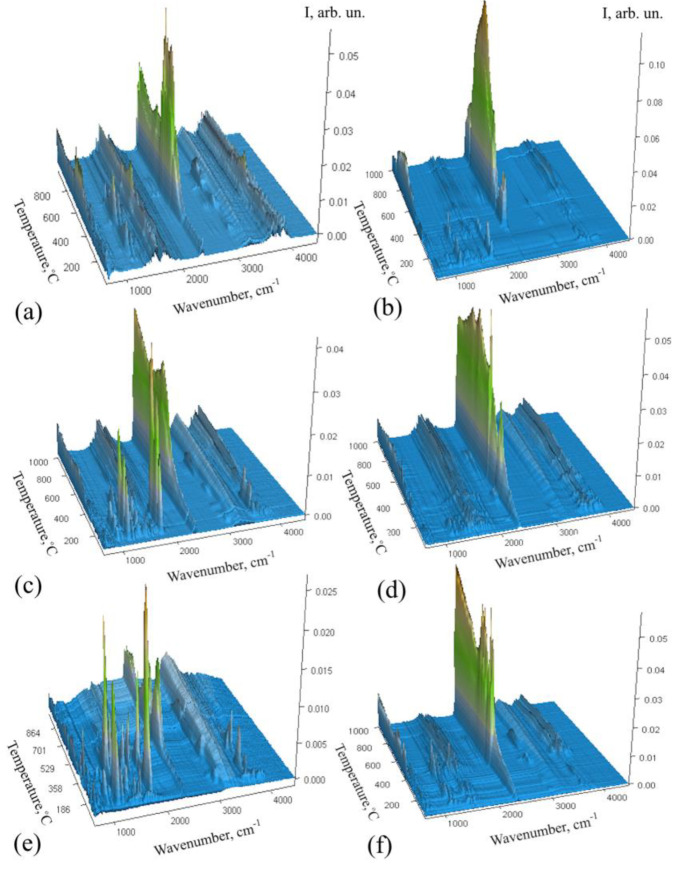
Fourier transform infrared spectrograms obtained during the decomposition of (**a**) PFR and composites: (**b**) PFR/SiO/Co, (**c**) PFR/SiO/Cu, (**d**) PFR/SiO/Ni, (**e**) PFR/SiO/Zn, (**f**) PFR/SiO.

Thermogravimetric analysis, coupled with Fourier transform infrared spectroscopy (TG–FTIR), was used to analyze the gasses released during the heat treatment of the polymer composite, which in turn facilitates the assessment of the structure and composition of the samples obtained (Figure 2). Different metal fillers alter the emission of volatile compounds prior to the degradation of the phenolic network. However, catalytic pyrolysis is very selective, and in a nitrogen environment, almost only hydrocarbons are produced.

At a temperature above 400 °C, the intense decomposition phase started, and mainly CO_2_ (699 and 2329–2358 cm^−1^), water vapor (above 3500 cm^−1^), and CO (2110–2184 cm^−1^) were emitted [39]. The detection of gasses in the 500–900 °C range showed a significant presence of volatile compounds (Figure 2), indicating the degradation of phenolic structures. They reached their highest intensity at 800 °C in PFR/SiO/Co, at 600 and 800° C in PFR/SiO/Cu, in the range of 650–850 °C in PFR/SiO/Ni, and at 650 and 800 °C in PFR/SiO. The PFR/SiO/Zn sample shows the lowest intensity of peaks that occurred at around 400, 550, and 850 °C. All these data correspond to the endothermic peaks on the DSC curves. Co-containing resins showed a higher number of volatile compounds over a wide temperature range, while the opposite trend was observed for Zn-containing resins, with very low CO_2_ emission intensity during pyrolysis. The appearance of very small bands at 2300 cm^−1^ during thermal degradation of the sample can be seen in Figure 2.

The different metals thus influenced the relative quantities and types of gasses released. However, in the samples containing Cu and Zn, a characteristic band of C=O stretching of formaldehyde at 1747 cm^−1^ was observed at low temperatures. The appearance of specific bands in the FTIR spectrum, such as those from 1050 to 1210 cm^−1^ (C–O stretching vibrations) and 1378–1446 cm^−1^ (deformation vibrations of –CH_3_ and –CH_2_ groups) during the thermal transformation of acetate salts and PF polymer can be attributed to the chemical interactions and decomposition processes that occur when the materials are heated. The decomposition of acetates releases acetic acid or acetate fragments, which can lead to the formation of acetoxy (CH_3_COO) groups that influence the decomposition of the polymer and contribute to the appearance of C–O stretching bands. The phenol–formaldehyde polymer contains phenolic hydroxyl groups (–OH), which can thermally transform and form new C–O bonds. These could be in the form of ethers (C–O–C), alcohols (C–OH), or ester-like structures as the polymer decomposes, leading to the C–O stretching vibrations observed in this region. In addition, the acetate groups decompose upon heating and form methyl (–CH_3_) and methylene (–CH_2_) fragments. It should be noted that alkyl chains (–CH_2_ or –CH_3_ groups) can also form during the thermal degradation of the PF resin as a result of cracking in the polymer. The breaking of the polymer chains leads to the release of small hydrocarbons or fragments containing these groups, which appear as bands in the range 1378–1446 cm^−1^ (Figure 2, PFR/SiO sample). However, in the presence of salts, this complex process starts at lower temperatures and is more intense. The sample containing Ni was not taken into account because nickel (II) nitrate hexahydrate was used.

In view of the above result, it can be assumed that the main volatile products are H_2_O, aromatic and aliphatic hydrocarbons, CO_2_, and CO. Catalytic pyrolysis has a high selectivity, and in a pure nitrogen environment, almost only hydrocarbons are produced. The thermal stability, degradation rate, and gas evolution during the pyrolysis of PF resins therefore vary, depending on the metal present.

### 2.2. Structural Evolution of Nanocomposites Revealed by XRD

**Figure 3:**
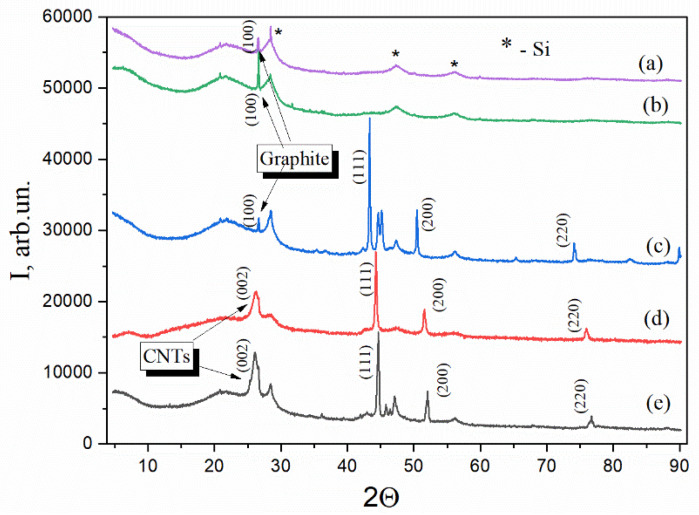
X-ray diffraction patterns for the composites C/SiO (a), C/SiO/Zn (b), C/SiO/Cu (c), C/SiO/Co (d), and C/SiO/Ni (e).

The crystallized structures of the nanocomposites were examined using XRD. This revealed significant structural changes that were influenced by the incorporated metals (Figure 3). A conspicuous broad hump observed in all samples within the range of 20–30° indicates the presence of amorphous carbon formed from disordered graphitic domains and non-crystalline silicon phases. The diffraction patterns display characteristic peaks at approximately 28.4°, 47.3°, and 56.2°, which are assigned to the (111), (220), and (311) planes of silicon (Si), indexed by JCPDS card No. 27-1402. Distinctive features are also observed in metal-containing composites. Crystalline SiO_2_ in its quartz form is evidenced by the appearance of peaks at 21°, 22°, and 26.6°, and a weaker peak around 20.8° in some samples. Additionally, peaks at 35.36° and 47.26° in the Ni- and Cu-containing samples may correspond to smaller crystalline phases or silica polymorphs. A broad peak at 25–26° in the C/SiO/Ni and C/SiO/Co samples associated with the (002) plane of graphitic carbon confirms the presence of carbon nanotubes (CNTs, JCPDS card No. 41-1487). This peak is less pronounced in CNT-containing samples, due to their lower crystallinity, than in highly ordered graphite, which typically exhibits a sharper, more pronounced peak at 26.5° (002) and a smaller peak around 42–44° (relative to the (100) plane). Thus, the peaks in the range of 42.36–43.26° are attributed to CNTs or graphitic (100) planes, further highlighting the structural contributions of carbon nanostructures in the metal-containing composites. CNTs are known for their large surface area and unique porous structure, which provide a variety of active sites for adsorption [40].

The crystalline nanostructure of the aforementioned metals was analyzed in detail. As illustrated in Figure 3, the XRD pattern of the Ni-containing composite exhibits prominent peaks at 2θ values of 44.5°, 51.8°, and 76.3°, which correspond to planes (111), (200), and (220) of crystalline metallic Ni, as indexed by JCPDS card No. 04-0850. The XRD analysis further confirmed the presence of distinct metallic phases in the samples. The Co-containing sample exhibited characteristic peaks at 2θ values of 44.2°, 51.5°, and 75.9°, corresponding to the (111), (200), and (220) planes of the face-cantered cubic (fcc) structure of metallic cobalt, as indexed by JCPDS card No. 15-0806. Similarly, the Cu-containing sample showed reflections at 43.3° (111), 50.4° (200), and 74.1° (220), indicating pure metallic copper with an fcc crystal structure, as indicated by JCPDS card No. 04-0836.

Interestingly, the zinc-containing sample showed no detectable reflections in its XRD pattern that correlate with ZnO crystals. This observation is consistent with previous studies indicating that the crystalline phase of zinc or zinc oxide decreases after high-temperature treatment during pyrolysis. The reduction of the ZnO phase to metallic zinc, driven by the presence of carbon at high temperatures, facilitates the evaporation of zinc in the form of metal vapor, which explains its absence in the analysis after pyrolysis [41,42]. The results obtained are confirmed by the results of the elemental analysis of the SEM study (data provided below).

### 2.3. Characterization of Carbon Nanostructures via Raman Spectroscopy

**Figure 4:**
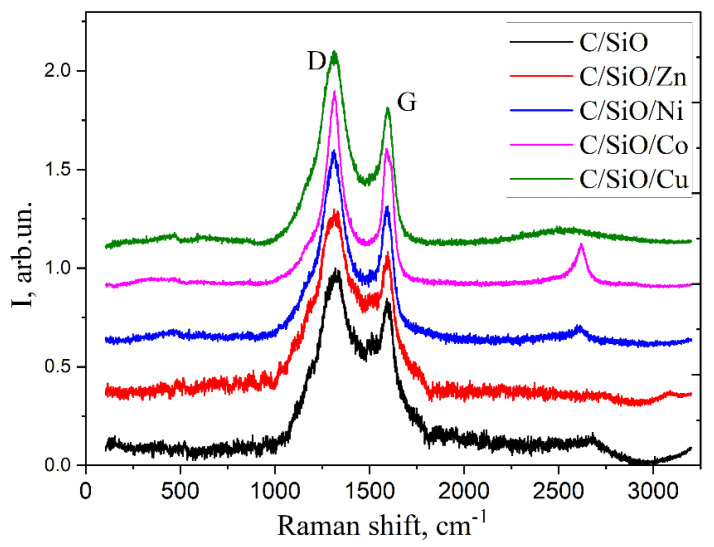
Raman spectra showing the positions of G-, D-bands for the studied composites.

In Figure 4, the Raman spectra are compared for the synthesized composites. The G band appears around 1580 cm^−1^ in the Raman spectrum of the graphitic carbon materials and is associated with the E2g phonon mode of the sp^2^-hybridized carbon atoms. It represents the symmetric stretching of all sp^2^ carbon atoms and reflects the in-plane vibrations of graphene or graphite [31]. In contrast, the D band observed at 1350 cm^−1^ is a vibration caused by defects in the sp^2^ carbon lattice. The 2610 cm^−1^ peak in carbonaceous materials, especially in the presence of metals, is often a second-order Raman feature associated with the 2D band or the overtone of the D band. The 2D band typically appears between 2600 and 2700 cm^−1^ and is more pronounced in carbon composites with metals.

Cobalt can enhance the peak at 2610 cm^−1^ in carbon composites because it amplifies the Raman signals through plasmonic effects or changes the electronic and structural properties of the carbon. Cobalt nanoparticles, when dispersed in a carbon matrix, can amplify the Raman signal by increasing the local electric field [32]. This is similar to what happens with copper or other transition metals such as gold and silver, which are commonly used in Surface-Enhanced Raman Spectroscopy (SERS).

The larger the full width at half maximum (FWHM) of the D peak in the Raman spectrum, the higher the degree of disorder or the amorphous character of the carbon material [43,44]. As can be seen from Table 2, the samples containing Zn and Cu together with SiO have a more disordered structure.

The I_G/D_ ratio evaluates the degree of disorder and crystallinity in the carbon structure. A high ratio indicates a high degree of graphitization or crystallinity, which means that the carbon material has fewer defects and a more ordered sp^2^ carbon network. This is typical for graphite and graphene, where the G-band (1580 cm^−1^, in-plane vibrations of sp^2^ carbon atoms) dominates over the D-band (corresponding to defects such as edge sites, vacancies, or non-hexagonal ring structures). In our series, the quality of the carbon materials decreases in terms of the defect density and degree of graphitization in the Co > Ni > Cu > Zn > SiO line. Co has been shown to reduce the activation energy (temperature ≤ 1000 °C) required for graphitization, producing well-structured carbons at relatively low temperatures.

### 2.4. Textural Properties

**Figure 5:**
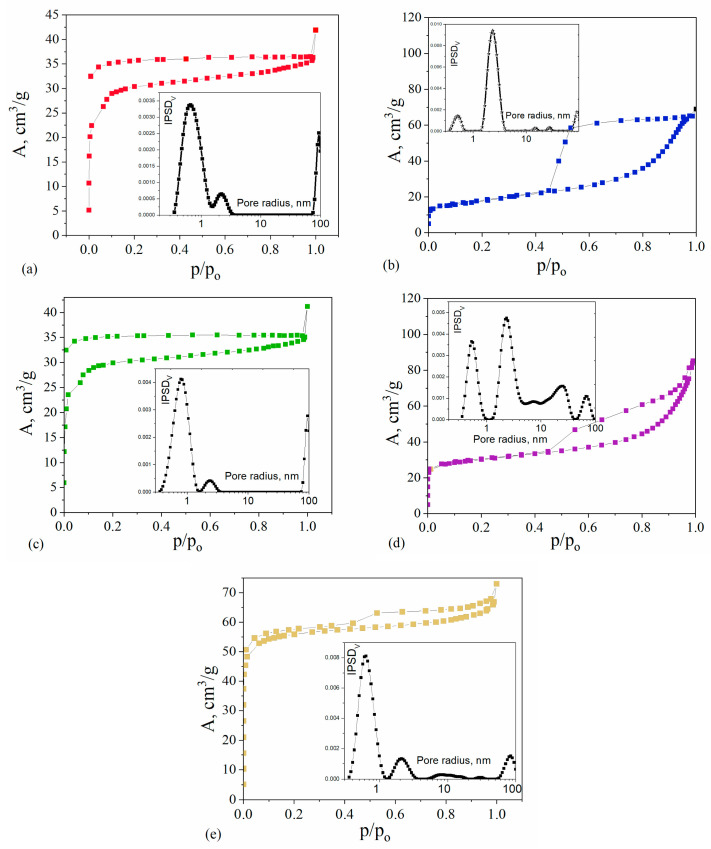
Low-temperature adsorption–desorption isotherms of nitrogen and the incremental pore size distributions (IPSDs) for the composites: C/SiO (**a**), C/SiO/Co (**b**), C/SiO/Cu (**c**), C/SiO/Ni (**d**), C/SiO/Zn (**e**).

The textural properties of the synthesized composites were determined based on nitrogen adsorption–desorption isotherms, which are shown in Figure 5. The nitrogen isotherms for all the studied composites correspond to type IV, according to the IUPAC classification, which is typical of the materials containing mesopores. However, the shape of the hysteresis loop indicates the formation of two types of mesopores: ink bottles for C/SiO/Co and cylindrical for the other materials. Two composites, C/SiO/Co and C/SiO/Ni, show relatively low adsorption at low relative pressures, p/po, and high adsorption at high pressures, indicating that their pore system is mainly composed of mesopores. For the remaining composites, C/SiO/Cu, C/SiO/Zn, and C/SiO, the sharp increase in nitrogen adsorption at low pressure indicates that micropores predominate in their structure. This can be confirmed by comparing the pore size distribution functions, which are also shown in Figure 5. For all composites, well-resolved peaks representing different types of pores are found, confirming the formation of two pore populations: micropores and mesopores of different sizes.

Table 3 shows the values of the texture parameters that characterize the different porosities of the obtained materials. The C/SiO composite, which was synthesized without the addition of metals, is characterized by low porosity. The total pore volume is 0.06 cm^3^/g, and mainly micropores can be recognized. On the other hand, the C/SiO/Cu composite shows a similar type of internal pore system, which proves that copper does not activate the formation of the developed porosity. For the composites synthesized with the addition of Co and Ni, an increase in the total pore volume is observed. The C/SiO/Co material is characterized by a large amount of mesopores, while the C/SiO/Ni composite material contains large amounts of micro- and mesopores. In contrast, the pore system of C/SiO/Zn and C/SiO/Cu composites consists mainly of micropores. The increased surface area and open mesoporous architecture can facilitate the rapid mass transfer of contaminants from the aqueous phase to the adsorptive sites and thus increase the adsorption of pollutants from the water.

### 2.5. SEM Analysis of Surface Texture and Microstructure

**Figure 6:**
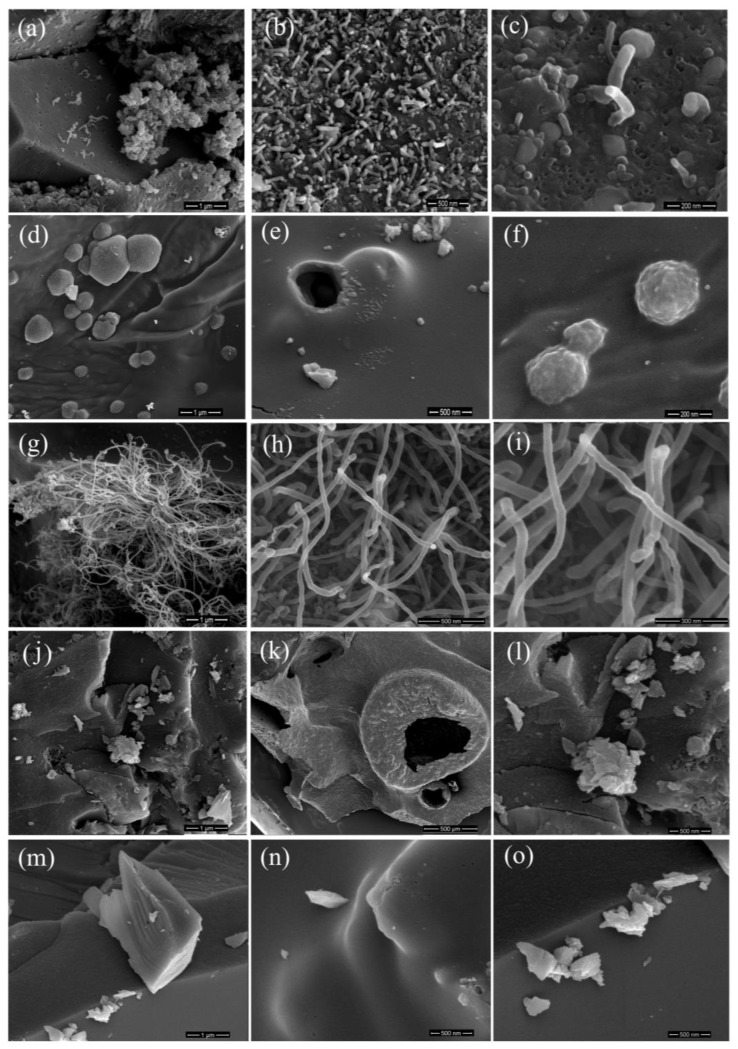
SEM micrographs for C/SiO/Co (**a**–**c**), C/SiO/Cu (**d**–**f**), C/SiO/Ni (**g**–**i**), C/SiO/Zn (**j**–**l**), and C/SiO (**m**–**o**).

Figure 6 shows scanning electron micrographs that allow a comparison of the morphological properties of the composites. As can be seen, the composites are characterized by diverse morphology. The samples are mostly composed of irregular particles, indicating that the ambiguous microstructure was not maintained. The microstructure of the C/SiO sample consists mainly of the carbon in an amorphous state; no graphite-like structures were observed in the phenolic resin sample without a catalyst agent. Compared to C/SiO, the Cu- and Co-containing samples exhibit a spherical microstructure, confirming the presence of the nucleation of crystalline carbon. The surface of the Zn composite is denser and smoother, while the surface of the Ni composite is more perforated and cracked.

It was also found that the addition of catalysts led to the formation of multi-walled carbon nanotubes (MWCNTs) in situ. As can be seen in Figure 6, a highly graphitized material with a carbon nanotube structure is formed in the pyrolytic C/SiO/Co and C/SiO/Ni samples at 950 °C, in contrast to the results published by Jiaming Liu et al. [45], where nanotube formation in the Ni-containing samples occurred at 1100 °C.

It is known that in the absence of metal, higher temperatures (above 1500 °C) are usually required to form well-structured forms of carbon [18]. The graphitization temperature can be significantly reduced by these metal catalysts. For example, Ni can promote the formation of graphitic carbon at 800–900 °C [22]. Ni is known to promote the growth of carbon nanofibers or carbon nanotubes during pyrolysis as it acts as a nucleation site for carbon atoms, resulting in highly structured carbon forms after treatment at 700 °C [46]. Co also promotes the formation of carbon nanotubes and graphitic carbon [47]. Cu has lower catalytic activity for graphitization compared to Ni or Co but can promote the formation of ultra-thin graphite [22,48]. Zn is less commonly used to promote graphitization but can help as a co-catalyst in the production of activated carbon or porous carbon structures. During pyrolysis, Zn tends to promote structural ordering of the deposited coke and reduce its hydrogen content [49].

The EDX-SEM analysis shows that the composites have different elemental compositions, with the contents of C, O, Si, and metals varying in the different samples (Table 4). The samples C/SiO/Co, C/SiO/Cu, and C/SiO/Ni show metal inclusions (~8 wt%) and a consistent Si presence around 9 wt%. In the case of the C/SiO/Zn composite, the lack of detectable metal (Zn) in the elemental composition is likely due to the evaporation of zinc during the synthesis process, which is consistent with the XRD data described previously. It is noteworthy that the Zn-containing composite (C/SiO/Zn) has the highest carbon content (88.65 wt%), while the Co- and Ni-containing samples contain more oxygen and silicon, indicating possible interactions with the carbon matrix and catalytic effects during pyrolysis.

### 2.6. Adsorption Properties

**Figure 7:**
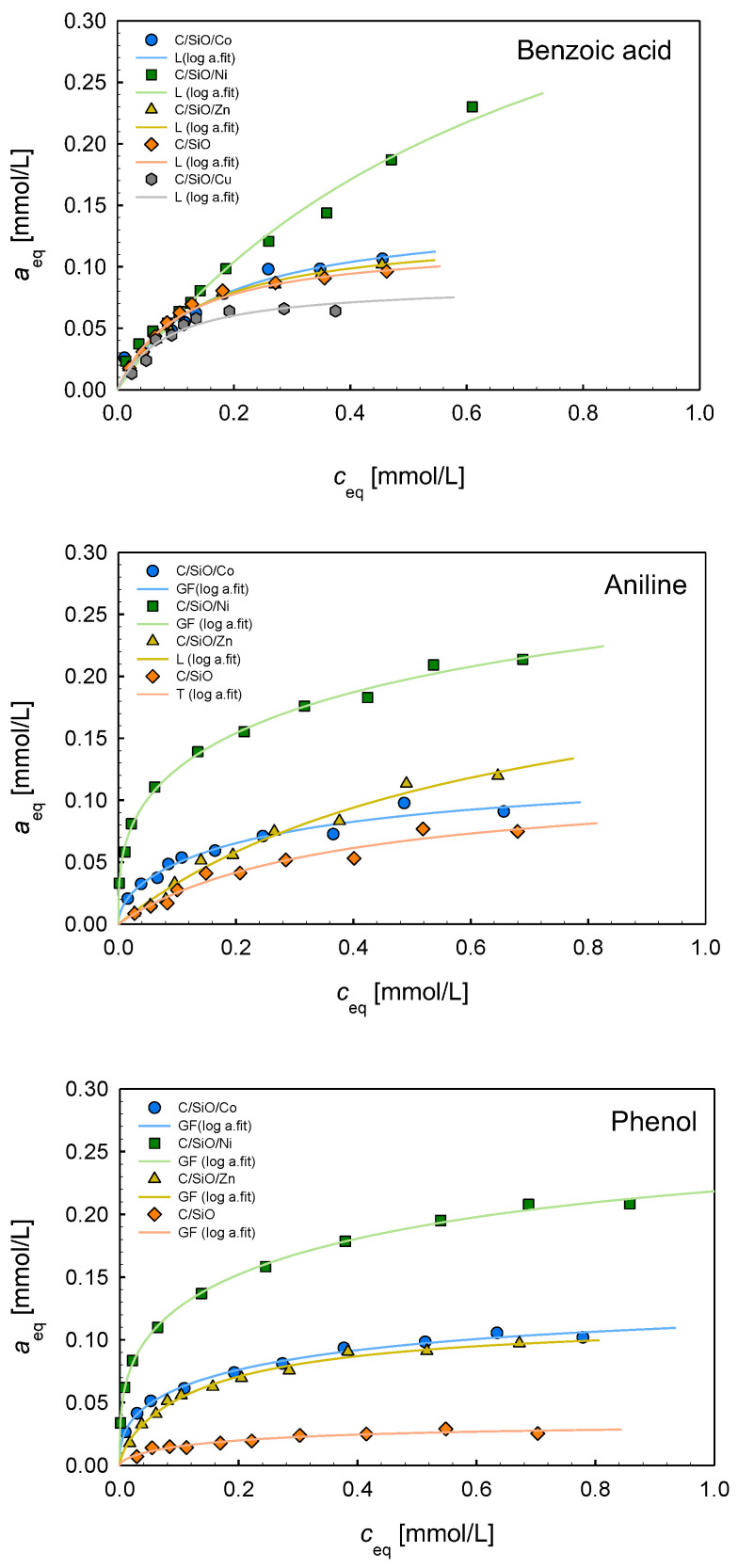
Comparison of adsorption isotherms of the composites C/SiO/Co, C/SiO/Ni, C/SiO/Zn, C/SiO, and C/SiO/Cu (only for benzoic acid) for benzoic acid, aniline, and phenol.

To analyze the influence of metal additives on the adsorption processes, the measurements of adsorption isotherms of aniline, benzoic acid, and phenol were conducted from aqueous solutions. In Figure 7, the adsorption isotherms of three adsorbates on the composites C/SiO/Co, C/SiO/Ni, C/SiO/Zn, and C/SiO are presented. The isotherms obtained for the C/SiO/Cu material are not shown because of very low adsorption effectiveness (only for benzoic acid does this material show affinity comparable with some other composites). Analyzing the experimental isotherms, the highest adsorption efficiency can be found in the case of the C/SiO/Ni composite for all adsorbates. The remaining materials show lower/significantly lower affinity to organic molecules. In the case of aniline and phenol, the isotherms can be arranged according to decreasing adsorption as follows: C/SiO/Co~C/SiO/Zn > C/SiO. For benzoic acid, this order is as follows: C/SiO/Co~C/SiO/Zn~C/SiO > C/SiO/Cu. To find correlations between composite properties and adsorption efficiency, the textural characteristics must first be taken into account. The most effective C/SiO/Ni material is characterized by S_BET_ = 103 m^2^/g, the largest pore volume (V_p_ = 0.132 cm^3^/g) consisting of meso- and macropores. The C/Si/Co composite, showing weaker adsorption affinity to organic solutes, has the specific surface area comparable to the C/Si sample (S_BET_ = 61 m^2^/g) and a lower pore volume (V_p_ = 0.107 cm^3^/g) consisting mainly of mesopores. Very well-developed porosity with a large population of micropores was found in the C/SiO/Zn composite (S_BET_ = 189 m^2^/g, V_p_ = 0.113 cm^3^/g). Thus, there is no simple relationship between the textural properties and adsorption capacity, indicating an important role of other factors. On the other hand, the composites C/Si and C/Si/Cu are characterized by very poor porosity and show weak affinity to organic solutes; it is especially observed for the material containing copper.

The other factors that should be considered are structural and morphological characteristics and the influence of metal active surface groups. As was evidenced by Raman spectra, the degree of graphitization decreases as follows: Co > Ni > Cu > Zn > SiO. The addition of nickel and cobalt promoted graphitization and the formation of carbon nanotubes, providing more ordered carbon structures. Such ordered carbon structures may enhance the adsorption of hydrophobic substances due to π–π interactions of the carbon surface with the benzene rings of the adsorbates. The composites synthesized with zinc addition have enhanced surface hydrophilicity, which may improve the adsorption of polar molecules.

**Figure 8:**
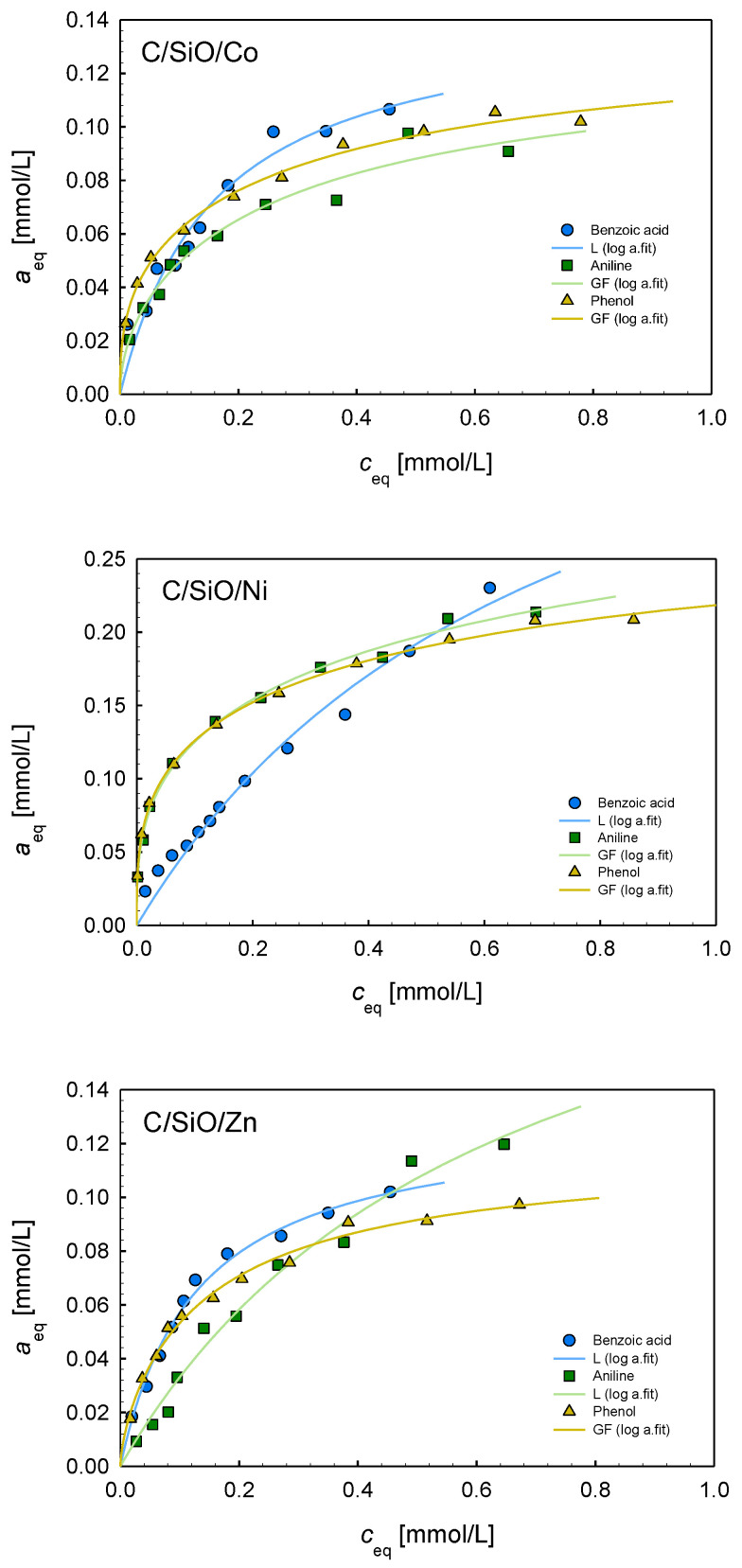

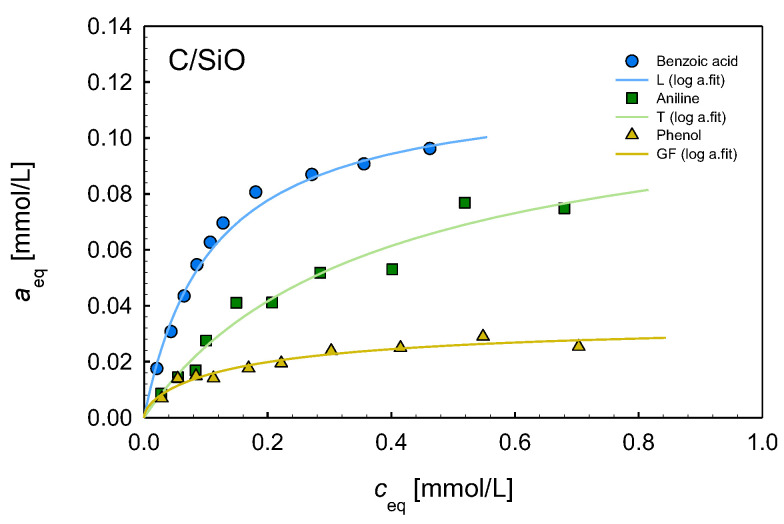
Comparison of adsorption isotherms of benzoic acid, aniline, and phenol from aqueous solutions on the composites C/SiO/Co, C/SiO/Ni, C/SiO/Zn, and C/SiO.

In Figure 8, the adsorption isotherms of three adsorbates are compared for the composites C/SiO/Ni, C/SiO/Co, C/SiO/Zn, and C/SiO. The greatest differentiation of isotherms is observed for the material C/SiO without metal addition. The highest adsorption is found for benzoic acid, lower for aniline, and the lowest for phenol. Such a tendency is very well correlated with their hydrophobic properties (solubilities): benzoic acid has the lowest solubility in water (c_s_ = 2.9 g/l at 20 °C), aniline is better solubilized (c_s_ = 3.7 g/l at 20 °C), while phenol is characterized by quite good solubility in water (c_s_ = 82 g/l at 20 °C), which decreases its affinity to the hydrophobic carbon surface. Thus, in the case of C/SiO material, the hydrophobic π–π interactions between the carbon surface and the benzene rings of adsorbates dominate in these systems. Meanwhile, in the case of the composites containing Ni, Co, and Zn, the adsorption isotherms for benzoic acid, aniline, and phenol show a similar course, confirming the previous statement about the important role of metallic active surface sites and the share of different adsorption mechanisms: π–π interactions; hydrogen bonding; interactions of adsorbate polar groups with adsorbent surface centers.

To analyze the adsorption equilibrium data, the Generalized Langmuir (GL) Equation (2) (Marczewski–Jaroniec, M-J isotherm) [50] was used in a multiparameter nonlinear fitting procedure. In Table 5, the parameters of this isotherm are compared for all adsorption systems, and the specific forms of the GL isotherm are provided (L, GF, T). The fitting quality is very good, as confirmed by the values of the standard deviation SD and determination coefficient R^2^. In the case of benzoic acid adsorption on all studied composites and aniline adsorption on C/SiO/Zn, homogeneity of the systems was found (m = *n* = 1, Langmuir isotherm). For other adsorption systems, the values of heterogeneity parameters, m and *n*, indicate moderate or strong heterogeneity effects. Moreover, one can state that the values of the adsorption capacity are in good agreement with the experimental maximum adsorption values.

Based on the values of the adsorption capacity (Table 5), the experimental systems may be ranged as follows: for benzoic acid, C/SiO/Ni (a_m_ = 0.45) > C/SiO/Co (a_m_ = 0.14)~C/SiO/Zn (a_m_ = 0.13)~C/SiO (a_m_ = 0.12) > C/SiO/Cu (a_m_ = 0.08); for aniline, C/SiO/Ni (a_m_ = 0.33) > C/SiO/Zn (a_m_ = 0.19) > C/SiO/Co (a_m_ = 0.12)~C/SiO (a_m_ = 0.11); for phenol, C/SiO/Ni (a_m_ = 0.28) > C/SiO/Co (a_m_ = 0.13)~C/SiO/Zn (a_m_ = 0.12) > C/SiO (a_m_ = 0.03). It is evident that the composite containing nickel is the most effective adsorbent for all the studied adsorbates of differentiated hydrophobicity and polarity. It is characterized by the largest total pore volume (micro- and mesopores). As the poorest adsorbents, C/SiO/Cu and C/SiO materials are characterized by small porosity. However, even a three- to four-fold increase in adsorption capacity with a simultaneous two-fold increase in pore volume for the C/SiO/Ni composite in comparison to the C/SiO/Cu and C/SiO materials underlines the important role of surface metallic centers.

## 3. Materials and Methods

### 3.1. Chemicals

Silicon monoxide (SiO, Fluka AG, Buchs, Switzerland); Copper (II) acetate hydrate and Zinc acetate dihydrate (ACS reagent, purity of ≥98%, Sigma-Aldrich, Poznań, Poland); Cobalt (II) acetate tetrahydrate and Nickel (II) acetate tetrahydrate (ACS reagent, purity of ≥98%, Merck, Poznań, Poland); and phenol–formaldehyde resin, PFR (novolac, SF-0112, “UkrKomCenter”, Kremenchuk, Ukraine), were used in the synthesis of composites. In the adsorption experiment, aniline, benzoic acid, and phenol delivered by Merck (Darmstadt, Germany) were used as adsorbates.

### 3.2. Preparation of Nanocomposites

The synthesis of nanocomposites consists of two steps. In the first step, silicon monoxide was mechanochemically mixed with an estimated weight of the corresponding metal acetate and PFR in a laboratory planetary ball mill PM 100 (Retsch GmbH, Haan, North Rhine-Westphalia, Germany), with agate balls and the vessel at a grinding speed of 350 rpm for 2 h. The content of the metals was 3.0 mmol/g SiO. In the second step, all powders obtained were calcined in air at 900 °C for 2 h. The reference sample without metal fillers was treated in the same steps. The polymer composites were labelled as PFR/SiO/Co, PFR /SiO/Cu, PFR /SiO/Ni, PFR /SiO/Zn, and PFR /SiO.

The carbonization of the samples was carried out in a tube furnace under a nitrogen atmosphere (with a flow rate of 100 mL/min) by heating from room temperature to 950 °C at a heating rate of 5 °C/min and holding at the maximum temperature for 2 h. The composites thus synthesized were designated as C/SiO/Co, C/SiO/Cu, C/SiO/Ni, C/SiO/Zn, and C/SiO.

### 3.3. Characterization Methods

X-ray diffraction (XRD) analysis was conducted by applying an Empyrean diffractometer (Malvern, PANalytical, Almelo, Netherlands) in reflection-transmission mode. As the radiation source (X-ray wavelength, 1.5418 Å), the X-ray tube with a Cu anode was used, and the PIXcel3D detector was applied in linear (1D) scanning mode. The samples were scanned over a 2θ range of 10–90°.

The Raman spectra were recorded using an inVia Reflex Microscope DMLM Leica Research Grade, Reflex, Renishaw, Wotton-under-Edge, Gloucestershire, UK (excitation at 514 nm).

The surface morphology of the samples was studied by field emission scanning electron microscopy (SEM, QuantaTM 3D FEG (FEI Company, Hillsboro, OR, USA), operating at a voltage of 30.0 kV).

To investigate the textural characteristics, low-temperature (77.4 K) nitrogen adsorption–desorption isotherms were recorded using a Micromeritics ASAP 2405N adsorption analyzer (Micromeritics Instrument Corporation, Norcross, GA, USA). The parameters characterizing the texture of the synthesized composites were determined from the measured isotherms by using the standard and specific methods [51,52,53]. The specific surface area (S_BET_) was calculated by using the BET method, and the total pore volume (V_p_) was obtained from the nitrogen adsorption value at the pressure p/p_o_~0.99. The incremental pore size distributions (IPSDs) were calculated using a model with slit-shaped cylindrical pores and voids between nanoparticles (SCV) [52,53]. The contributions of micropores (micropore surface area, S_mic_, and micropore volume, V_mic_), mesopores (mesopore surface area, S_mes,_ and mesopore volume, V_mes_), and macropores (macropore surface area, S_mac,_ and macropore volume, V_mac_) were calculated from these distribution functions.

Thermal decomposition of samples was determined using a STA 449 Jupiter F1 (Netzsch, Selb, Bavaria, Germany) coupled online with an FTIR spectrometer (Bruker, Ettlingen, Baden-Württemberg, Germany). The samples (~12 mg) were heated at a rate of 10 °C/min in the range of 30–1000 °C in the atmosphere of nitrogen (50 mL/min) in an alumina crucible and sensor thermocouple type S TG–DSC. An empty Al_2_O_3_ crucible was used as a reference. The data were recorded and processed using NETZSCH Proteus^®^ software, version 6.1.

### 3.4. Adsorption from Solutions

The adsorption isotherms from dilute aqueous solutions were measured for aniline, benzoic acid, and phenol as adsorbates. The first stage of the applied procedure [54] was as follows: a sample of composite was contacted with a solution of adsorbate of determined concentration, and after attaining the adsorption equilibrium (2–3 days), the solute concentration was measured by using the UV-Vis spectrophotometer Cary 4000 (Varian, Melbourne, Victoria, Australia). In the next steps to each adsorption system, a certain portion of concentrated adsorbate was added, and after attaining the new equilibrium, the adsorbate concentration was measured. The procedure was repeated to measure the adsorption isotherms over a wide range of concentrations. The solute adsorbed amount was calculated from the mass balance:

(1) $$a_{eq}=\frac{\left(c_{o}-c_{eq}\right)\cdot V}{m}$$

where *a_eq_*—the amount of substance adsorbed on the adsorbent at equilibrium (mmol/g), *c_o_*—initial solution concentration (mmol/l), *c_eq_*—concentration of the solution at equilibrium (mmol/l), *V*—solution volume (dm^3^), and *m*—adsorbent mass (g).

The generalized Langmuir (GL) equation [50], which describes well the systems characterized by energetic inhomogeneity, was used to analyze the experimental adsorption isotherms:

(2) $$\theta ={\left[\frac{{\left(Kc_{eq}\right)}^{n}}{1+{\left(Kc_{eq}\right)}^{n}}\right]}^{m/n}$$

where *θ* = *a_eq_*/*a_m_*—relative adsorption of a solute in the surface phase; a_m_—adsorption capacity; *m* and *n*—heterogeneity parameters characterizing the shape (width and asymmetry) of the adsorption energy distribution function and taking values from the range (0,1); and *K*—equilibrium constant describing the position of the distribution function on the energy axis.

For specific values of the heterogeneity parameters m and *n*, the GL equation reduces to the known adsorption isotherms:

- when the heterogeneity parameters take the values m = *n* ϵ (0,1), we obtain the Langmuir–Freundlich (LF) isotherm equation;
- when the heterogeneity parameters take the values *n* = 1 and m ϵ (0,1), we obtain the Generalized Freundlich (GF) isotherm equation;
- when the heterogeneity parameters take the values m = 1 and *n* ϵ (0,1), we obtain the Tóth (T) isotherm equation;
- when the heterogeneity parameters take the values m = *n* = 1, we obtain the Langmuir (L) isotherm equation.

## 4. Conclusions

The carbonization of novolac phenol–formaldehyde resins in the presence of SiO, and Co, Cu, Ni, and Zn improves the structure, morphology, and thermal stability of the resulting carbon materials. Nickel and cobalt are particularly effective in promoting graphitization and the formation of carbon nanotubes, while copper and zinc improve the porosity and surface area of the carbon. The presence of metals during the thermal transformation of phenol–formaldehyde polymers can significantly influence the decomposition process, leading to the formation of different carbon structures. Metal fillers, especially Co and Ni, likely act as catalysts during decomposition, promoting char formation and altering heat release patterns.

No simple relationship was observed between textural (specific surface area, pore volume) and structural (degree of graphitization) properties and adsorption capacity. The important role of metallic surface groups was evidenced in the adsorption efficiency and mechanism of the process. In the case of nickel and cobalt, a higher degree of graphitization and more ordered carbon structures can promote adsorption of hydrophobic substances due to π–π interactions. The zinc-containing composites with enhanced surface hydrophilicity can facilitate adsorption of polar molecules. For C/SiO material, the predominance of hydrophobic π–π interactions was found, while for other composites, a mixed adsorption mechanism was evidenced: π–π interactions, hydrogen bonding, and interactions with metallic surface centers. Moreover, pore structure and accessibility play important roles. Co- and Ni-containing composites, even with a fewer number of formed CNTs in C/SiO/Co, have a pore structure that favors the adsorption of relatively small organic molecules. If the pores are optimally sized for the adsorption of these pollutants, the effective adsorption can be high even when the overall surface area is lower.

These findings demonstrate that the proposed technology offers a cost-effective method for recycling PFR to produce carbon composites with enhanced structural and functional properties. Notably, the resulting composites exhibit improved carbon nanostructures and retention of crystalline silicon. Furthermore, the incorporation of Co, Cu, Ni, or Zn into novolac resin-derived carbon materials enhances their physicochemical and structural characteristics, making them highly advantageous for future applications in fields such as adsorption, catalysis, and energy storage. Research will continue to increase the porosity, efficiency, and selectivity of composites by modifying the synthesis conditions and introducing various sources of organic carbon.

## Figures and Tables

**Table 1:** Thermal behavior of composite samples (T_max_ is the maximum degradation temperature determined for DTG and DSC curves, Δm—is the total weight loss).

Sample	Temperature Range °C	Mass Loss %	Δm_total_ %	T_max_^DTG^ °C	T_max_^DSC^; (Endo↑; Exo↓) °C
PFR	36–116	1.02	62.87	85.9	87.4↑
	116–205	3.22		183.2	168.7↑
	205–315	9.60		283.5	
	315–495	29.05		383.5	393.2↑; 445.8↑
	495–626	11.34		548.1	555.4↑
	626–1000	8.64		-	878.7↑
PFR/SiO	36–120	0.96	45.54	86.9	70.3↑
	120–306	7.69		258	166↑
	306–384	8.33		364.9	373.7↑; 381.7↑
	384–462	7.84		402.8	406.8↑
	462–1000	20.72		483.5; 516.6; 548.6	473.3↑; 867↑
PFR/SiO/Co	36–120	4.69	61.47	91.7	95.1↑
	120–192	3.06		152.9	157.9↑
	192–312	6.31		295.6	301↑; 320.9↑
	312–440	8.28		321	385.9↑
	440–542	7.86		499.5	456↑
	542–881	28.72		609.6; 633.2	547.1↑
	881–1000	2.55		949.3	761.5↑
PFR/SiO/Cu	36–130	4.71	35.64	106.6	110.9↑
	130–185	4.13		144.5	
	185–293	5.76		218.1	189↑; 218.1↑
	293–491	7.65		386.2	381.4↑; 422.5↑
	491–1000	13.39		590.6	857.8↑
PFR/SiO/Ni	36–98	2.77	41.98	82.3	82.3↑
	98–152	3.80		114.1	124.9↑
	152–202	3.08		174.8	
	202–268	3.58		233.4	
	268–352	6.23		314.8	294.5↑; 318.9↑
	352–514	7.08		455.5	458↑
	514–1000	15.44		633.6	667.8↑
PFR/SiO/Zn	36–80	1.71	33.49	57.1	58.8↑
	80–204	6.21		120.5;151.8	150.7↑; 165.7↑
	204–471	15.36		309.4	309↑; 426.7↑
	471–821	8.53		499.4	505↑
	821–1000	1.68		888.7	847.5↑

**Table 2:** Structural characteristics of all studied composites.

Sample	D	G	2D	FWHM _D_	FWHM _G_	IG/D (Area Integral)
C/SiO/Co	1308.41	1594.3	2617	69.1	62.2	(0.97)
C/SiO/Cu	1309.71	1593.1		119.6	63.5	(0.59)
C/SiO/Ni	1305.51	1590.2	2610	101.1	63.2	(0.85)
C/SiO/Zn	1299	1592.2		131	70.2	(0.43)
C/SiO	1302	1592		121.4	67.7	(0.45)

**Table 3:** Values of parameters characterizing the porosity of the studied composites.

Sample	S_BET_ m^2^/g	S_micro_ m^2^/g	S_meso_ m^2^/g	S_macro_ m^2^/g	V_p_ cm^3^/g	V_micro_ cm^3^/g	V_meso_ cm^3^/g	V_macro_ cm^3^/g	V_micro_/V_p_	V_meso_/V_p_
C/SiO/Co	61	20.5	40.3	0.1	0.108	0.010	0.090	0.008	0.090	0.834
C/SiO/Cu	95	86.6	8.4	0.1	0.063	0.045	0.009	0.009	0.721	0.139
C/SiO/Ni	103	71.4	31.1	0.4	0.132	0.032	0.086	0.014	0.246	0.651
C/SiO/Zn	189	179.7	9.0	0.2	0.112	0.085	0.017	0.010	0.755	0.154
C/SiO	105	96.6	8.7	0.1	0.063	0.045	0.010	0.008	0.710	0.157

**Table 4:** Elemental composition of the composites based on the EDX-SEM study.

	Element Composition (wt%)				
Sample	C	O	Si	Metal	Total
C/SiO/Co	74.5	7.78	9.15	8.57	100
C/SiO/Cu	81.93	3.17	6.93	7.97	100
C/SiO/Ni	73.89	8.13	9.15	8.83	100
C/SiO/Zn	88.65	5.13	6.22	-	100
C/SiO	79.27	11.50	9.23	-	100

**Table 5:** The parameters of the Generalized Langmuir (GL) isotherm equation characterizing adsorption of benzoic acid, aniline, and phenol on the studied materials.

Composite	Isotherm Type	*a_m_*	*m*	*n*	log *K*	*R* ^2^	SD*(a)*
Benzoic acid							
C/SiO/Co	L	0.14	1	1	0.79	0.941	0.007
C/SiO/Ni	L	0.45	1	1	0.14	0.979	0.009
C/SiO/Zn	L	0.13	1	1	0.89	0.989	0.003
C/SiO	L	0.12	1	1	0.96	0.977	0.005
C/SiO/Cu	L	0.08	1	1	1.06	0.928	0.005
Aniline							
C/SiO/Co	GF	0.12	0.51	1	0.24	0.963	0.005
C/SiO/Ni	GF	0.33	0.32	1	−0.32	0.996	0.004
C/SiO/Zn	L	0.19	1	1	0.20	0.982	0.006
C/SiO	T	0.11	1	0.95	0.44	0.958	0.005
Phenol							
C/SiO/Co	GF	0.13	0.36	1	0.07	0.992	0.002
C/SiO/Ni	GF	0.28	0.29	1	−0.17	0.998	0.003
C/SiO/Zn	GF	0.12	0.65	1	0.63	0.990	0.003
C/SiO	GF	0.03	0.56	1	0.48	0.931	0.002

*a_m_*—adsorption capacity [mmol/g]; *m*, *n*—heterogeneity parameters; *K*—equilibrium constant related to characteristic adsorption energy; *R*^2^—determination coefficient; SD—standard deviation.

## Data Availability

Data are available on request from the corresponding author.
